# Supplementary material for: Ionization of Decamethylmanganocene: Insights from the DFT-Assisted Laser Spectroscopy
Source: Molecules. 2022 Sep 22;27(19):6226. doi: 10.3390/molecules27196226 (PMC9573365; doi:10.3390/molecules27196226)
Supplement: Supplementary file 1 [file molecules-27-06226-s001.zip › C5Me5_2_Mn_xyz_revised.pdf]

Table S1. Atomic coordinates of the optimized $Cp^*_2Mn^0$ molecule.

Atom	X	Y	Z
C	-1.5989472	-0.8811340	-1.0317232
C	-1.5676230	0.5335740	-1.2838460
C	-1.7708316	1.2032249	-0.0337184
C	-1.9291553	0.2139791	0.9782771
C	-1.8219361	-1.0689567	0.3705632
C	1.6009283	0.8370753	1.0755420
C	1.8257201	1.0971151	-0.3131697
C	1.9286171	-0.1511503	-0.9882289
C	1.7665440	-1.1932444	-0.0295888
C	1.5653516	-0.5882822	1.2549857
C	1.4939328	-1.3096991	2.5660339
H	0.9147880	-0.7491005	3.3042073
H	1.0285610	-2.2930239	2.4613003
H	2.4947790	-1.4661627	2.9890512
C	1.8863830	-2.6615529	-0.2976199
H	2.9253073	-2.9985637	-0.1896451
H	1.2823733	-3.2490531	0.3982168
H	1.5655556	-2.9169200	-1.3105212
C	2.1970514	-0.3389567	-2.4485639
H	3.2754231	-0.3928959	-2.6440990
H	1.7529448	-1.2632319	-2.8259307
H	1.8003239	0.4871968	-3.0434472
C	1.9906291	2.4487514	-0.9354484
H	1.6743179	2.4552643	-1.9814081
H	1.4095787	3.2097316	-0.4089008
H	3.0405996	2.7669848	-0.9105137
C	1.5564789	1.8623356	2.1667548
H	2.5611416	2.0702552	2.5571309
H	1.1426745	2.8106865	1.8146608
H	0.9448438	1.5289018	3.0087474
C	-1.9930954	-2.3860012	1.0617303
H	-3.0447702	-2.6996610	1.0554498
H	-1.4175288	-3.1763039	0.5735039
H	-1.6736957	-2.3409612	2.1057503
C	-2.1961457	0.4780023	2.4270244
H	-3.2744471	0.5375398	2.6212965
H	-1.7954253	-0.3143625	3.0638501
H	-1.7551149	1.4224970	2.7544651
C	-1.5615403	-1.9609887	-2.0693279
H	-1.1421166	-2.8891079	-1.6728955
H	-2.5689066	-2.1906413	-2.4401583
H	-0.9578769	-1.6697816	-2.9325746
C	-1.4912048	1.1865902	-2.6300135
H	-0.9010384	0.5937738	-3.3333371
H	-2.4897544	1.3122305	-3.0683672

H	-1.0350500	2.1781409	-2.5725775
C	-1.8814588	2.6838055	0.1603227
H	-1.5490981	2.9882452	1.1558720
H	-1.2824840	3.2327112	-0.5703756
H	-2.9201419	3.0193900	0.0463841
Mn	-0.0001581	-0.0012281	-0.0002019

Table S2. Atomic coordinates of the optimized Cp*$_2$Mn$^+$ cation.

Atom	X	Y	Z
C	-1.6257141	-0.8823114	-1.0349435
C	-1.5948631	0.5342809	-1.2873876
C	-1.7937261	1.2065579	-0.0346950
C	-1.9496565	0.2147676	0.9815767
C	-1.8440572	-1.0720366	0.3708925
C	1.6278887	0.8384518	1.0789933
C	1.8479973	1.1006173	-0.3133516
C	1.9486718	-0.1514948	-0.9915515
C	1.7889379	-1.1961782	-0.0287097
C	1.5926384	-0.5888258	1.2585039
C	1.5139820	-1.3089226	2.5674854
H	0.9474670	-0.7454269	3.3110724
H	1.0547988	-2.2939387	2.4661996
H	2.5204496	-1.4600313	2.9734702
C	1.9053933	-2.6626210	-0.2955021
H	2.9488603	-2.9792249	-0.1869377
H	1.3151797	-3.2545816	0.4063272
H	1.5923479	-2.9224964	-1.3082222
C	2.2188918	-0.3393157	-2.4487920
H	3.3000113	-0.3903042	-2.6208703
H	1.7884497	-1.2670109	-2.8298968
H	1.8358524	0.4882758	-3.0483017
C	2.0125746	2.4504652	-0.9335278
H	1.7069095	2.4615238	-1.9811911
H	1.4449243	3.2173977	-0.4036062
H	3.0666712	2.7482820	-0.9002749
C	1.5771907	1.8616147	2.1689688
H	2.5870486	2.0561220	2.5471674
H	1.1741305	2.8137346	1.8191787
H	0.9744622	1.5281422	3.0155975
C	-2.0132880	-2.3875752	1.0600011
H	-3.0690264	-2.6810753	1.0466629
H	-1.4522294	-3.1836030	0.5671861
H	-1.7022800	-2.3471657	2.1053404
C	-2.2188709	0.4781516	2.4272226
H	-3.3000144	0.5329975	2.5980293
H	-1.8315922	-0.3153480	3.0687275

H	-1.7920926	1.4263855	2.7585084
C	-1.5803940	-1.9603912	-2.0710363
H	-1.1673287	-2.8907592	-1.6771343
H	-2.5928762	-2.1799099	-2.4278895
H	-0.9886888	-1.6675448	-2.9402087
C	-1.5112792	1.1857602	-2.6315578
H	-0.9312101	0.5909370	-3.3394146
H	-2.5152962	1.3041465	-3.0541127
H	-1.0638631	2.1798795	-2.5774397
C	-1.9031188	2.6850502	0.1575854
H	-1.5821171	2.9947131	1.1537409
H	-1.3162213	3.2385872	-0.5775106
H	-2.9464022	2.9993023	0.0407413
Mn	-0.0002190	-0.0010158	-0.0001945

Table S3. Calculated and experimental selected bond lengths ***r*** in the $Cp^*{}_2Mn^0$ molecule.

Bond	$r_{calc.}$	$r_{exp.}$[a]	$r_{exp.}$[b]
C1-C2	1.424	1.421	1.431
C2-C3	1.433	1.433	1.402
C3-C4	1.437	1.411	1.424
Mn-C1	2.174	2.117	2.127
Mn-C2	2.142	2.111	2.104
Mn-C3	2.095	2.107	2.098

[a] Ref.1; [b] Ref.2.

Table S4. Calculated and experimental selected angles ϕ (°) in the $Cp^*{}_2Mn^0$ molecule.

Angle	$\phi_{calc.}$	$\phi_{exp.}$[a]	$\phi_{exp.}$[b]
Mn-C1-C6	178.8	178.5	178.2
Mn-C2-C7	177.4	177.8	177.6
Mn-C3-C8	174.9	177.1	177.0
Mn-C4-C9	174.9	177.8	178.2
Mn-C5-C10	177.3	177.6	179.3

[a] Ref.1; [b] Ref.2.

References

1. Freyberg, D.P.; Robbins, J.L.; Raymond, K.N.; Smart J.C. Crystal and molecular structures of decamethylmanganocene and decamethylferrocene. Static Jahn-Teller distortion in a metallocene. *J. Am.Chem. Soc.* **1979**, *101*, 892–897. DOI: 10.1021/ja00498a017.

2. Augart, N.; Boese, R.; Schmid G. Phasenübergang von Decamethylmanganocen. *Z. Anorg. Allg. Chem.* **1991**, *595*, 27–34. DOI: 10.1002/zaac.19915950105.
