# Supplementary figures and images for: Ionization of Decamethylmanganocene: Insights from the DFT-Assisted Laser Spectroscopy
Source: Molecules. 2022 Sep 22;27(19):6226. doi: 10.3390/molecules27196226 (PMC9573365; doi:10.3390/molecules27196226)

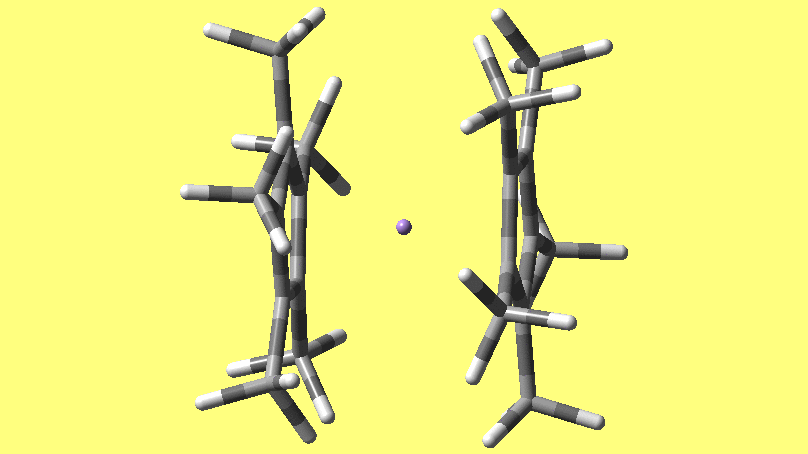

Supplement: Supplementary file 1 [file molecules-27-06226-s001.zip › Video S1_vib_130.gif]

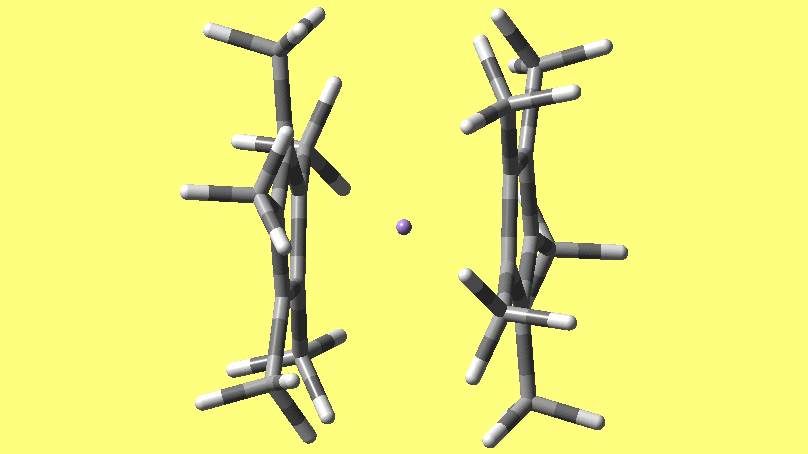

Supplement: Supplementary file 1 [file molecules-27-06226-s001.zip › Video S2_vib_352.gif]
